# Associations Between Industry Payments to Physicians for Antiplatelet Drugs and Utilization of Cardiac Procedures and Stents

**DOI:** 10.1007/s11606-021-06980-6

**Published:** 2021-08-10

**Authors:** Mao Yanagisawa, Daniel M. Blumenthal, Hirotaka Kato, Kosuke Inoue, Yusuke Tsugawa

**Affiliations:** 1 Division of General Internal Medicine and Health Services Research, David Geffen School of Medicine at UCLA, Los Angeles, CA USA; 2 Cardiology Division, Department of Medicine, Massachusetts General Hospital, Boston, MA USA; 3 Harvard Medical School, Boston, MA USA; 4 Coeur Value, LLC, Wellesley, MA USA; 5 Graduate School of Business Administration, Keio University, Yokohama, Japan; 6 Department of Epidemiology, UCLA Fielding School of Public Health, Los Angeles, CA USA; 7 Department of Health Policy and Management, UCLA Fielding School of Public Health, Los Angeles, CA USA

## Abstract

**Background:**

A study has shown that industry payments to physicians for drugs are associated not only with higher drug prescriptions but also with higher non-drug costs due to additional utilization of healthcare services. However, the association between industry payments to cardiologists for antiplatelet drugs and the costs and number of percutaneous coronary interventions they perform has not been investigated.

**Objective:**

To examine the association between industry payments to cardiologists for antiplatelet drugs and the costs and number of percutaneous coronary interventions they perform.

**Design:**

Using the 2016 Open Payments Database linked to the 2017 Medicare Provider Utilization and Payment Data, we examined the association between the value of industry payments related to the antiplatelet drugs prasugrel and ticagrelor and healthcare spending and volume for cardiovascular procedures, adjusted for potential cofounders.

**Subjects:**

A total of 7456 cardiologists who performed diagnostic cardiac catheterizations on Medicare beneficiaries in 2017.

**Main Measures:**

Primary outcomes included (1) healthcare spending on cardiac procedures, (2) diagnostic cardiac catheterization volumes, and (3) rates of coronary stenting. Secondary outcomes were total expenditures for all drugs and for antiplatelet drugs.

**Key Results:**

Industry payments for antiplatelet drugs were associated with higher healthcare spending on cardiac procedures (adjusted difference, +$50.9 for additional $100 industry payments; 95% CI, +$25.5 to +$76.2; P < 0.001), diagnostic cardiac catheterizations (+0.1 procedures per cardiologist; 95% CI, +0.03 to +0.1; P=0.001), and stent use (+0.5 per 1000 diagnostic cardiac catheterizations per cardiologist; 95% CI, +0.2 to +0.9; P=0.002). Industry payments for antiplatelet drugs were associated with higher total costs for all drugs and antiplatelet drugs.

**Conclusions:**

Industry payments to cardiologists for antiplatelet drugs were associated with both prescribing of antiplatelet drugs and the use of cardiac procedures and stents. Further research is warranted to understand whether the observed associations are causal or reflect a greater propensity for higher volume proceduralists to have relationships with industry.

**Supplementary Information:**

The online version contains supplementary material available at 10.1007/s11606-021-06980-6.

## INTRODUCTION

The physician-industry relationship has been shown to create conflicts of interests, which can distort physicians’ prescription patterns of drugs and medical devices, and may adversely impact clinical outcomes.^1–7^ However, little is known as to whether industry payments for drugs influence physicians’ clinical decision-making other than utilization of the targeted drug. One recent study found a positive association between industry payments and non-drug-related healthcare spending, suggesting that industry payments for drugs may have a dynamic impact on physicians’ behavior that extends beyond the prescription practice.^8^ Yet, although industry payments may exert such “off-target” spillover effects, the mechanisms as to which physician practices (other than prescriptions) are affected by them remain unclear. This knowledge gap has hindered our understanding of the broader implications of industry payments to physician behavior, and efforts to develop policies that could effectively restrict influences of industry on physicians’ practice patterns.

In this study, we examined the association between industry payments for antiplatelet drugs and physicians’ practice of cardiac procedures. We hypothesized that industry payments for antiplatelet drugs might distort not only physicians’ prescription practices but also physicians’ likelihood of performing cardiac procedures including stent placement, after which patients receive antiplatelet drugs.^9^ Passed by Congress in 2010, the Physician Payments Sunshine Act required that all manufacturers of drugs, devices, biologicals, or medical supplies file annual reports of payments to clinicians via Open Payments, a publicly available database managed by the Centers for Medicare & Medicaid Services (CMS). We used the Open Payments database linked with the national database of utilization and payments for procedures and prescription drugs to examine the association between industry payments to physicians for new antiplatelet drugs and the use of cardiac procedures and coronary stents.

## METHODS

### Data

We linked five publicly available databases managed by CMS: (1) the 2016 Open Payments data, (2) the 2017 Medicare Provider Utilization and Payment Data: Physician and Other Supplier (MPOS), (3) the 2017 Medicare Provider Utilization and Payment Data: Part D Prescriber, (4) the Physician Compare database, and (5) the National Plan & Provider Enumeration System (NPPES) database.

#### Industry Payments

Payments made by manufacturers and group purchasing organizations are reported through the Open Payments data in three categories: general payments (e.g., food and beverage, travel and lodging, speaker compensation, consulting fees, and education), research payments (e.g., funding for research), and physician ownership information (e.g., ownership and investment interest in companies). We extracted data on general payments for antiplatelet drugs and coronary stent products. In this study, we defined “industry payments” as payments made for antiplatelet drugs to physicians by pharmaceutical companies. We identified industry payments for three P2Y12 inhibitors recommended for patients after coronary stent placement as antiplatelet drugs (clopidogrel, prasugrel, and ticagrelor).^9^

#### Procedure Utilization and Drug Prescription by Physicians

The MPOS database includes information on utilization and costs for services provided to Medicare beneficiaries by healthcare providers.^10^ The Part D database provides information on drugs prescribed by healthcare providers under the Medicare Part D Prescription Drug Program.^11^ To protect the privacy of Medicare beneficiaries, the MPOS data excludes practices representing fewer than or equal to 10 beneficiaries by a physician, and the Part D data removes the data on prescriptions containing fewer than or equal to 10 claims by a physician. We identified the claims made by physicians for diagnostic cardiac catheterization using the Current Procedural Terminology (CPT) code 93454 to 93461, and the claims for stent placement using the CPT code of 92928, 92929, 92933, 92934, 92937, 92938, 92941, 92943, and 92944. The total expenditures for each procedure were calculated based on the sum of the Medicare payment amount, the deductible, and coinsurance amounts that the beneficiaries were responsible for paying, and any amounts that a third party was responsible for paying. The utilization of diagnostic cardiac catheterizations and percutaneous coronary stent placement was defined as the number of services provided to Medicare beneficiaries. The total expenditures for drugs were defined as the amount paid by the Medicare Part D plan, the Medicare enrollee, and other third-party payers or government subsidies; the ingredient cost of the medication; dispensing fees; sales tax; and any applicable administration fees.

#### Physician Characteristics

The Physician Compare database provides general information on individual physicians and other clinicians including gender, medical school graduation year, and medical school attended.^12^ Years since graduation from medical school were categorized into four groups: ≤10 years, 11–20 years, 21–30 years, and >30 years. Medical schools were categorized into three groups based on research ranking of US medical schools reported in the 2017 US News & World Report: top 20, 21–50, and all other schools, including unranked and foreign medical schools. Physician specialty was categorized using the NPPES database.

### Data Linkage

The National Provider Identifier (NPI), a unique identifier for physicians, was used to link the MPOS data, the Part D data, and the Physician Compare data. Given that the Open Payments data does not include NPI, we used physicians’ full name and the zip code for the primary practice location in the NPPES database ^13^ to link this database with other data, an approach used in the previous studies.^14,15^

### Outcomes Studied

Primary outcomes were (1) healthcare costs (defined as Medicare allowed amount) for cardiac procedures (the sum of diagnostic cardiac catheterization and stenting), (2) the utilization of diagnostic cardiac catheterization per cardiologist, and (3) the frequency of stent placement per 1000 diagnostic cardiac catheterizations per cardiologist. Secondary outcomes were costs for (1) all drugs and (2) the antiplatelet drugs.

### Physician Inclusion and Exclusion Criteria

**Figure 1:**
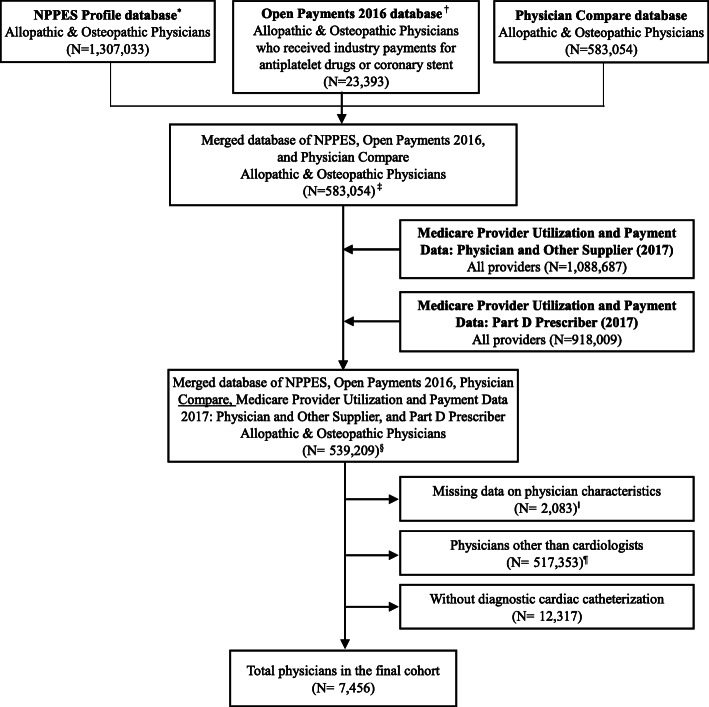
Flow diagram of database merging and selection process. ^*^National Plan and Provider Enumeration System (NPPES) file was accessed in February 2020. ^†^Open Payment database 2016 originally had more than 10 million observations. We converted them to the dataset for each physician using the physician profile ID. ^‡^Concatenated string of “First name_Last name_Zip code” was used to merge NPPES and Open Payments. ^§^In the merged dataset, 17,966 physicians received industry payments for antiplatelet drugs or coronary stent in 2016. ^‖^Data on graduation year was missing among 2083 physicians. Data on medical school attended, and physician gender was not missing. ^¶^Cardiologists were identified using the Health Care Provider Taxonomy Code.

The main analysis in this study included all physicians with at least eleven claims for diagnostic cardiac catheterizations in the MPOS data and whose specialty was cardiology (see Fig. 1 for more details). We excluded physicians with missing data on key characteristics (physician gender, years in practice, and medical school attended). We also excluded physicians with industry payments or outcomes above the 99th percentile in the value of industry payments and outcome measures to minimize the effects of outliers in illustrating correlations between industry payments for antiplatelet drugs and primary outcomes.

### Adjustment Variables (Potential Confounders)

We adjusted for physician characteristics (physician gender, years in practice, and the research ranking of the medical school attended), the amount of industry payments for coronary stents (per $100), the number of beneficiaries, average Hierarchical Condition Category (HCC) risk scores of Medicare beneficiaries served by each physician (extracted from the MPOS data), and state fixed effects.

### Statistical Analysis

We investigated associations between industry payments and each primary and secondary outcome measure, after adjustment for potential confounders. We used ordinary least squares (OLS) regression models with Huber-White heteroscedasticity-robust standard errors.

### Sensitivity Analyses

We conducted 10 sensitivity analyses by restricting our sample to physicians who performed at least 11 diagnostic cardiac catheterizations and stent insertion procedures in 2017. First, to account for potential misclassification of physician specialty (some cardiologists may be coded as “internal medicine”), we included all physicians who performed diagnostic cardiac catheterizations and stent insertion procedures, regardless of their specialty as listed in the NPPES. Second, we limited our sample to interventional cardiologists. Third, to test the robustness of our findings based on sample selection, we restricted our sample to cardiologists who received payments for antiplatelet drugs (our main analyses included cardiologists receiving no industry payments). Fourth, we adjusted for the number of stent insertion procedures in 2015 to address the possibility that physicians who performed more stent insertion procedures were more likely to be targeted by the industry. Fifth, given that the industry may be targeting physicians who prescribed more antiplatelet drugs, we additionally adjusted for the number of prescribed antiplatelet drugs in the previous year (2015). Sixth, for a similar reason, we adjusted for the cost of prescribed antiplatelet drugs in the previous year (2015). Seventh, to examine whether our findings were sensitive to our selection of the regression models, we reanalyzed the data using negative binomial regression models. Eighth, we tested whether the results differ between the model with and without the average HCC risk score, by removing the average HCC risk score from adjustment variables and comparing its results with that of the analysis with the average HCC risk score. Ninth, we used years since physicians’ graduation as a continuous variable (with quadratic and cubic terms to account for possible non-linear relationship) instead of a categorical variable. Tenth, to account for the possible non-linear association between industry payments and outcomes, we analyzed the data using the value of industry payments for antiplatelet drugs as a categorical variable (categorized as $0, $1–$50, $51–$100, $101–$200, and >$200). Finally, to test whether industry payments have short-term impacts, we conducted a cross-sectional study by using both exposure and outcomes from the 2017 data.

All analyses were performed using R version 3.6.3. The study was exempted for review by the UCLA Office of the Human Research Protection Program, Institutional Review Board.

## RESULTS

We found that 0.4% of physicians identified in the merged dataset (NPPES, Open Payments (2016), Physician Compare, MPOS (2017), and Part D database (2017)) had the missing data on key characteristics (physician gender, years in practice, and medical school attended). Only two physicians received industry payments for clopidogrel. As clopidogrel had been approved over 10 years before the approval of prasugrel and ticagrelor and has been widely used, there may have been little incentive for clopidogrel manufacturers to make payments to physicians, and the payment for clopidogrel may have limited impact on physicians’ practice. Therefore, we excluded payments and prescription costs for clopidogrel from the analyses. Our final sample included 7456 physicians (Fig. 1).

### Physician Characteristics

General payments for antiplatelet drugs (prasugrel and ticagrelor) worth $3.8 million were made to 4269 cardiologists in our final sample treating Medicare beneficiaries in 2016. Physicians who received payments for antiplatelet drugs were more likely to be male and less likely to have graduated from US medical schools ranked in the top 20 for research by US News and World Report (Table 1).

**Table 1:** Physician characteristics by receipt of payments for antiplatelet drugs

	Overall	Received payments for antiplatelet drugs		P value
		No	Yes	
No.	7456	3187	4269	
	Mean (SD^*^)			
Total Medicare allowed amount: cardiac procedures, $	37,912 (38,721)	35,582 (39,491)	39,652 (38,047)	<0.001
Number of diagnostic cardiac catheterizations per cardiologist	79.0 (69.8)	74.8 (70.0)	82.2 (69.5)	<0.001
Number of stent use per 1000 diagnostic cardiac catheterizations per cardiologist	300.7 (316.2)	296.7 (333.0)	303.8 (303.1)	0.34
Total drug cost: all drugs, $	302,341 (349,619)	239,670 (336,559)	349,127 (351,895)	<0.001
Total drug cost: antiplatelet drugs, $	19,474 (28,808)	12,244 (22,067)	24,872 (31,905)	<0.001
Payments for antiplatelet drugs, $	514 (3823)	0 (0)	897 (5018)	<0.001
Payments for stent, $	366 (2419)	300 (2396)	416 (2436)	0.04
Number of beneficiaries	1266 (765)	1182 (770)	1330 (755)	<0.001
Beneficiary average HCC risk scores	1.89 (0.39)	1.90 (0.41)	1.89 (0.38)	0.69
	No. (%)			
Gender				0.001
Male	7131 (95.6)	3018 (94.7)	4113 (96.3)	
Female	325 (4.4%)	169 (5.3%)	156 (3.7%)	
Years in practice				0.43
≤10	345 (4.6%)	158 (5.0%)	187 (4.4%)	
11–20	1809 (24.3%)	749 (23.5%)	1060 (24.8%)	
21–30	2404 (32.2%)	1035 (32.5%)	1369 (32.1%)	
>30	2898 (38.9%)	1245 (39.1%)	1653 (38.7%)	
Medical school attended				<0.001
Top 20	715 (9.6%)	371 (11.6%)	344 (8.1%)	
Ranked 21–50	1135 (15.2%)	524 (16.4%)	611 (14.3%)	
Other schools^†^	5606 (75.2%)	2292 (71.9%)	3314 (77.6%)	

*Standard deviation

†Including all unranked and foreign medical schools

### Industry Payments and Healthcare Costs for Cardiac Procedures

**Figure 2:**
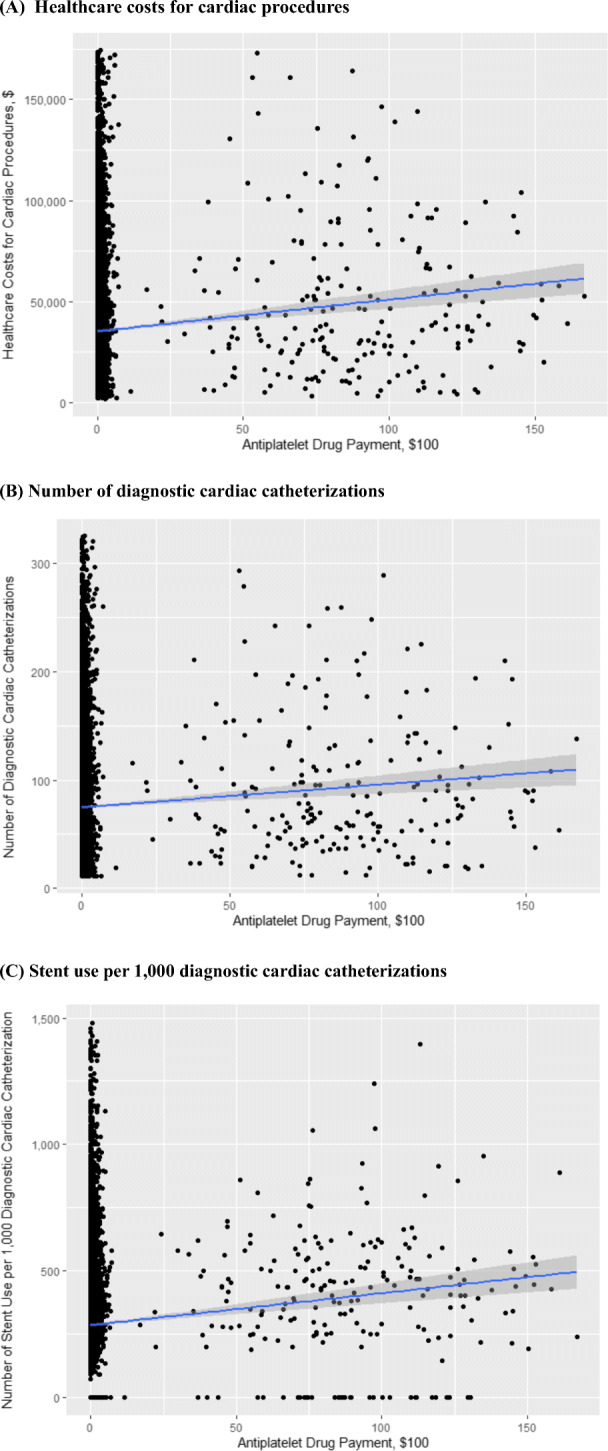
Correlation between industry payments for antiplatelet drugs and (**A**) healthcare costs for cardiac procedures, (**B**) number of diagnostic cardiac catheterizations, and (**C**) stent use per 1000 diagnostic cardiac catheterizations. (**A**) Linear regression model of healthcare costs on cardiac procedures and the value of payments for antiplatelet drugs for cardiologists below the 99th percentile in healthcare costs on cardiac procedures and the value of payments. The shaded area represents 95% CI. (**B**) Linear regression model of the utilization of diagnostic cardiac catheterizations per cardiologist and the value of payments for antiplatelet drugs for cardiologists below the 99th percentile in the use of diagnostic cardiac catheterizations and the value of payments. The shaded area represents 95% CI. (**C**) Linear regression model of the stent use per 1000 diagnostic cardiac catheterizations per cardiologist and the value of payments for antiplatelet drugs for cardiologists below the 99th percentile in the stent use per 1000 diagnostic cardiac catheterizations and the value of payments. The shaded area represents 95% CI.

Total healthcare costs for diagnostic cardiac catheterizations and coronary stent procedures by cardiologists in our final sample in 2017 were $283 million. We found a positive correlation between industry payment and total cardiac procedure costs (Fig. 2 (A)). After adjusting for potential confounders, industry payments to physicians for antiplatelet drugs were associated with higher cardiac procedure costs (adjusted difference, +$50.9 for additional $100 industry payments; 95% CI, +$25.5 to +$76.2; P < 0.001) (Table 2). Based on the $3.8 million of industry payments for antiplatelet drugs, extra spending for cardiac procedures by industry payments is estimated to be $1.9 million.

**Table 2:** Association between industry payments to physicians^*^ for antiplatelet drugs and the use of cardiac procedures, stents, and prescriptions (for $100 increase in industry payments)

	Mean	Adjusted difference (95% CI)		P value
Primary outcome				
Total healthcare costs for cardiac procedures, $	37,912	+50.9	(+25.5 to +76.2)	<0.001
Number of diagnostic cardiac catheterization per cardiologist, No.	79.0	+0.1	(+0.03 to +0.1)	0.001
Stent use per 1000 diagnostic cardiac catheterizations per cardiologist, No.	300.7	+0.5	(+0.2 to +0.9)	0.002
Secondary outcome				
Total spending on all drugs, $	302,341	+295.4	(+50.6 to +540.3)	0.02
Total spending on antiplatelet drugs, $	19,474	+66.6	(+30.8 to +102.5)	<0.001

*Physicians were restricted to cardiologists who performed at least 11 diagnostic cardiac catheterizations on Medicare beneficiaries in 2017 (N=7456)

### Industry Payments and Utilization of Diagnostic Cardiac Procedures and Coronary Stents

In 2017, 7456 cardiologists performed 588,983 diagnostic cardiac catheterizations on Medicare beneficiaries, and 4718 filed 214,935 claims for percutaneous coronary stent placement in Medicare patients. Total spending on diagnostic cardiac procedures and coronary stent placement totaled $160 million and $123 million, respectively. In unadjusted analyses, we found a positive correlation between industry payments for prasugrel and ticagrelor and the utilization of diagnostic cardiac procedures, and between industry payments and placement of coronary stents (Fig. 2 (B) (C)). After adjusting for potential confounders, industry payments for antiplatelet drugs were associated with a larger number of diagnostic cardiac catheterizations performed (adjusted difference, +0.1 procedures per cardiologist for additional $100 industry payments; 95% CI, +0.03 to +0.1; P=0.001) (Table 2). Similarly, in adjusted analyses, each additional $100 in industry payments for antiplatelet drugs was associated with increased stent use per 1000 diagnostic cardiac catheterizations (adjusted difference, +0.5 stent procedures per cardiologist for additional $100 industry payments; 95% CI, +0.2 to +0.9; P=0.002) (Table 2).

### Industry Payments and Drug Costs

The costs to Medicare for all prescribed drugs and antiplatelet drugs in 2017 by physicians in our final sample were $2.3 billion and $145 million, respectively. After adjusting for potential confounders, each additional $100 in industry payments for antiplatelet drugs was significantly associated with greater spending on all drugs (adjusted difference, +$295.4 per $100 increase in industry payments; 95% CI, +$50.6 to +$540.3; P = 0.02) and antiplatelet drugs (adjusted difference, +$66.6 per $100 increase in industry payments; 95% CI, +$30.8 to +$102.5; P < 0.001) (Table 2). Based on the $3.8 million of industry payments for antiplatelet drugs, extra spending for antiplatelet drugs by industry payments is estimated to be $2.5 million.

### Sensitivity Analyses

Our findings were qualitatively unaffected by including all physicians regardless of specialty, restricting to interventional cardiologists, restricting to interventional cardiologists who received payments for antiplatelet drugs, adjusting for the number of stent use in the prior year, adjusting for the number of prescribed antiplatelet drugs in the prior year, adjusting for the costs of prescribed antiplatelet drugs in the prior year, using negative binomial regression models, removing the average HCC risk score from adjustment variables, and using physicians’ practice years as a continuous variable with quadratic and cubic terms (Supplementary Table D-M). When we restricted our analyses to interventional cardiologists, we found that associations between industry payments for antiplatelet drugs and the use of diagnostic cardiac catheterizations and stent use were no longer statistically significant (diagnostic cardiac catheterizations: adjusted difference, +0.02 per $100 increase in industry payments; 95% CI, −0.01 to +0.1; P=0.14. Stent use per 1000 diagnostic cardiac catheterizations, +0.2 per $100 increase in industry payments; 95% CI, −0.1 to +0.6; P=0.13) (Supplementary Table F). The sample size was reduced to 4718 when we restricted our sample to interventional cardiologists, and the statistical power may also have decreased. When we used industry payments as a categorical variable, we found a monotonic increase in outcome measures for higher payment categories. For example, cardiologists who received >$200 industry payments for antiplatelet drugs spent $6878 more (95% CI, +$2987 to +$10,769; P<0.001) on cardiac procedures compared to cardiologists who received no industry payments (Supplementary Table N). In the cross-sectional study using industry payments in 2017 as exposure, associations between industry payments and the use of diagnostic cardiac catheterizations and stent and the spending on drugs were not statistically significant (Supplementary Table O). The substantial decrease in the total value of industry payments for prasugrel in our final sample from $221,803 in 2016 to $4397 in 2017, when generic prasugrel was approved, may explain the difference in the results between the cross-sectional analysis and our main analysis.

## DISCUSSION

Using the national database of industry payments to physicians, we found that industry payments for antiplatelet drugs were not only associated with higher prescriptions but also associated with increased costs of cardiac procedures and stents. In particular, industry payments for antiplatelet drugs were associated with greater healthcare costs for cardiac procedures, increased use of diagnostic cardiac procedures, and a higher likelihood of utilizing coronary stents. These findings raise the possibility that, if observed relationships were causal, industry payments to physicians not only distort their prescription practices but could also influence physicians’ decisions about the use of procedures, tests, and imaging studies that are indirectly associated with the prescription of drugs for which industry payments were made. Policymakers should be aware of the broad influence that industry financial relationships have on physician healthcare service utilization behavior, and better understand how these relationships may affect the quality and costs of care.

Industry payments may have the greatest effect on “gray zone” decision-making—situations in which a procedure or treatment could be useful, but is not clearly indicated or contra-indicated.^16,17^ Percutaneous coronary intervention (PCI) for patients with acute myocardial infarction has been shown to be associated with a 27% lower odds of patient mortality in randomized clinical trials.^18^ However, when used to treat stable angina or large areas of ischemia identified on stress testing among patients with stable coronary disease, PCI does not confer a mortality benefit, and may be no more effective at alleviating symptoms than optimal medical therapy.^19–22^ Regardless of the minimum benefit of PCI for those patient populations, there are concerns about the potential overuse of PCI for patients with stable coronary disease,^23^ which would expose patients to unnecessary risks, including complications after the procedure and side effects from subsequent medication, and increase medical costs for unnecessary procedures as well as treatments for complications.

Our study builds upon a recent study that has suggested that industry payments to physicians may be associated with non-drug-related costs. Meija and colleagues recently found that total industry payments were associated with increased non-drug-related medical costs.^8^ However, this study was limited as they investigated only aggregated total medical costs, and potential mechanisms as to how industry payments lead to increased non-drug costs have not been investigated. Moreover, the study did not adjust for physician characteristics such as physicians’ gender and clinical experience; therefore, it was possible that the observed relationship could be explained by the fact that certain type of physicians (e.g., male physicians, physicians with more clinical experience ^14,15,24^) are receiving higher industry payments and providing more intensive (and expensive) care. To our knowledge, this study is the first to adjust for physician-level confounders when evaluating associations between industry payments for medicines and non-drug-related healthcare service use and spending, with robust adjustment for a broad set of potential physician-level confounders. Our study should spur future research focused on understanding whether the associations observed in this study are, in fact, causal, whether clinical measures other than cardiac procedures are also associated with industry payments for antiplatelet drugs, how industry payments distort physician behaviors, and if and how they increase the likelihood of ordering tests and imaging studies to identify asymptomatic diseases that may lead to higher prescriptions of drugs marketed by industry.

Our study has limitations. First, as is the case with any observational studies, we could not eliminate the possibility of unmeasured confounding. For instance, our results could be overestimated if physicians who treat sicker patients and are thus more likely to undergo procedures were receiving a higher amount of industry payments in general. As our dataset is comprised of physician-level data, important patient characteristics, including comorbidity, clinical status, stability, and cardiac lesion features, which may affect physicians’ decision on cardiac procedural utilization, were not available. However, the results of our sensitivity analysis showed that the associations remained largely unchanged whether or not we adjusted for the average HCC risk score of beneficiaries treated by each physician, indicating that patients’ severity of illness probably is not a major confounder of this study. Second, it is also possible that the industry may be targeting key clinical opinion leaders, who are higher volume operators, rather than that industry payments are causing physicians to use more stents. In our main analysis, we used data on industry payments in 2016 as exposure variables and the utilization in 2017 as outcome variables to address the issues of reverse causality. We also adjusted for the number of prescriptions in 2015 for antiplatelet drugs by each physician in a sensitivity analysis. However, our study could not completely remove the possibility of reverse causality. Third, our analyses did not include physicians with fewer than 11 claims for cardiac procedures because data for these physicians were suppressed in the MPOS database. Therefore, our findings may not be generalizable to low-volume physicians with regard to the number of cardiac procedures performed. Fourth, our findings may not be generalizable to procedures other than those included in this study, or to non-Medicare populations. Finally, our study could not demonstrate that the associations between exposure and outcome were causal.

Using large national datasets, we found that industry payments made to physicians for antiplatelet drugs were associated with higher rates of prescribing antiplatelet drugs and higher non-drug medical costs for cardiac procedures resulting from the greater use of diagnostic cardiac catheterizations and coronary stenting. These findings raise the possibility that industry payments for physicians not only affect their prescribing behavior but also influence decisions about the use of invasive tests and procedures.

## Supplementary Information

ESM 1 (PDF 440  kb)

## References

[1] Sharma M, Vadhariya A, Johnson ML, Marcum ZA, Holmes HM (2018). Association between industry payments and prescribing costly medications: an observational study using open payments and medicare part D data. BMC Health Serv Res..

[2] DeJong C, Aguilar T, Tseng C-W, Lin GA, Boscardin WJ, Dudley RA (2016). Pharmaceutical Industry–Sponsored Meals and Physician Prescribing Patterns for Medicare Beneficiaries. JAMA Intern Med..

[3] Perlis RH, Perlis CS (2016). Physician Payments from Industry Are Associated with Greater Medicare Part D Prescribing Costs. PLoS ONE.

[4] Modi PK, Wang Y, Kirk PS, Dupree JM, Singer EA, Chang SL (2018). The Receipt of Industry Payments is Associated With Prescribing Promoted Alpha-blockers and Overactive Bladder Medications. Urology..

[5] Fujiwara RJT, Shih AF, Mehra S (2017). Cross-sectional Analysis of the Relationship between Paranasal Sinus Balloon Catheter Dilations and Industry Payments among Otolaryngologists. Otolaryngol Head Neck Surg..

[6] Eloy JA, Svider PF, Bobian M (2017). Industry relationships are associated with performing a greater number of sinus balloon dilation procedures. International Forum of Allergy & Rhinology..

[7] Annapureddy AR, Henien S, Wang Y (2020). Association Between Industry Payments to Physicians and Device Selection in ICD Implantation. JAMA..

[8] Mejia J, Mejia A, Pestilli F (2019). Open data on industry payments to healthcare providers reveal potential hidden costs to the public. Nat Commun..

[9] Levine GN, Bates ER, Blankenship JC (2011). 2011 ACCF/AHA/SCAI Guideline for Percutaneous Coronary Intervention. Journal of the American College of Cardiology..

[10] Medicare Provider Utilization and Payment Data: Physician and Other Supplier PUF CY2017 | Data.CMS.gov. Accessed May 5, 2020. https://data.cms.gov/Medicare-Physician-Supplier/Medicare-Provider-Utilization-and-Payment-Data-Phy/fs4p-t5eq

[11] Medicare Provider Utilization and Payment Data: 2017 Part D Prescriber | Data.CMS.gov. https://data.cms.gov/Medicare-Part-D/Medicare-Provider-Utilization-and-Payment-Data-201/77gb-8z53. Accessed 5 May 2020.

[12] Archived Datasets | Data.Medicare.gov. Data.Medicare.Gov. Accessed May 5, 2020. https://data.medicare.gov/data/archives/physician-compare

[13] NPI Files. Accessed May 5, 2020. https://download.cms.gov/nppes/NPI_Files.html

[14] Tringale KR, Marshall D, Mackey TK, Connor M, Murphy JD, Hattangadi-Gluth JA (2017). Types and Distribution of Payments From Industry to Physicians in 2015. JAMA..

[15] Inoue K, Blumenthal DM, Elashoff D, Tsugawa Y (2019). Association between physician characteristics and payments from industry in 2015–2017: observational study. BMJ Open..

[16] Chandra A, Skinner J (2012). Technology Growth and Expenditure Growth in Health Care. Journal of Economic Literature..

[17] Chandra A, Khullar D, Lee TH (2015). Addressing the Challenge of Gray-Zone Medicine. N Engl J Med..

[18] Fazel R, Joseph TI, Sankardas MA, et al. Comparison of Reperfusion Strategies for ST-Segment–Elevation Myocardial Infarction: A Multivariate Network Meta-analysis. *J Am Heart Assoc*. 2020;9(12). doi:10.1161/JAHA.119.015186 10.1161/JAHA.119.015186 PMC7429064 32500800

[19] Kereiakes DJ, Teirstein PS, Sarembock IJ (2007). The Truth and Consequences of the COURAGE Trial. J Am Coll Cardiol..

[20] McFalls EO, Krupski WC, Hattler B, Ellis N. Coronary-Artery Revascularization before Elective Major Vascular Surgery. *The New England Journal of Medicine*. Published online 2004:10. 10.1056/NEJMoa041905 15625331

[21] Maron DJ, Hochman JS, Reynolds HR (2020). Initial Invasive or Conservative Strategy for Stable Coronary Disease. New England Journal of Medicine..

[22] Boden WE, O’Rourke RA, Teo KK (2007). Optimal Medical Therapy with or without PCI for Stable Coronary Disease. New England Journal of Medicine..

[23] Weintraub WS, Weiss S, Bikak AL. Percutaneous Coronary Intervention for Stable Ischemic Heart Disease. In: Myat A, Clarke S, Curzen N, Windecker S, Gurbel PA, eds. *The Interventional Cardiology Training Manual*. Springer International Publishing; 2018:255-261. doi:10.1007/978-3-319-71635-0_18

[24] Brunt CS (2019). Physician Characteristics, Industry Transfers, and Pharmaceutical Prescribing: Empirical Evidence From Medicare and the Physician Payment Sunshine Act. Health Serv Res..

