# Supplementary material for: Associations Between Industry Payments to Physicians for Antiplatelet Drugs and Utilization of Cardiac Procedures and Stents
Source: J Gen Intern Med. 2021 Aug 10;37(7):1626–33. doi: 10.1007/s11606-021-06980-6 (PMC9130441; doi:10.1007/s11606-021-06980-6)
Supplement: Supplementary file 1 — (PDF 440 kb) [file 11606_2021_6980_MOESM1_ESM.pdf]

Supplementary Appendix

Associations between Industry Payments to Physicians for Antiplatelet Drugs and Utilization of Cardiac Procedures and Stents

Supplementary Table A. Characteristics of payments for antiplatelet drugs in 2016 to cardiologists who performed diagnostic cardiac catheterizations in 2017.

Nature of Payments	Number of payments	% of No.	Payment amount, $	% of Payment
**Food and Beverage**	29,698	91.1%	543,845	14.2%
**Travel and Lodging**	1,374	4.2%	309,950	8.1%
**Compensation for services other than consulting, including serving as faculty or as a speaker at a venue other than a continuing education program**	1,232	3.8%	2,812,425	73.4%
**Education**	229	0.7%	10,516	0.3%
**Consulting Fee**	80	0.2%	154,422	4.0%
**Total**	32,613	100%	3,831,157	100%

Supplementary Table B. Characteristics of payments for coronary stents in 2016 to cardiologists who performed diagnostic cardiac catheterizations in 2017

Nature of Payments	Number of payments	% of No.	Payment amount, $	% of Payment
**Food and Beverage**	16,816	84.3%	590,297	21.6%
**Travel and Lodging**	2,327	11.7%	851,552	31.2%
**Compensation for services other than consulting, including serving as faculty or as a speaker at a venue other than a continuing education program**	411	2.1%	658,386	24.1%
**Consulting Fee**	349	1.8%	624,965	22.9%
**Education**	37	0.2%	4,379	0.2%
**Total**	19,940	100%	2,729,578	100%

Supplementary Table C. Manufacturers making payments for coronary stents in 2016 to cardiologists who performed diagnostic cardiac catheterizations in 2017

Manufacturers	Number of payments	% of No.	Payment amount, $	% of Payment
**Abbott Laboratories**	9,412	47.2%	1,138,325	41.7%
**Medtronic Vascular, Inc.**	8,118	40.7%	1,491,815	54.7%
**Boston Scientific Corporation**	2,150	10.8%	67,578	2.5%
**Medinol USA Inc**	253	1.3%	31,318	1.1%
**Medtronic, Inc.**	7	0.04%	542	0.02%
**Total**	19,940	100%	2,729,578	100%

Supplementary Table D. Association between industry payments to physicians* for antiplatelet drugs and the use of cardiac procedures, stents, and prescriptions, for $100 increase in industry payments, including all physicians regardless of their specialty

	Mean	Adjusted Difference (95% CI)		P Value
**Primary outcome**				
**Total healthcare costs for cardiac procedures, $**	52,876	+27.3	(+8.0 to +46.6)	0.01
**Number of diagnostic cardiac catheterization per physician, No.**	103.3	+0.03	(-0.01 to +0.1)	0.11
**Stent use per 1,000 diagnostic cardiac catheterizations per physician, No.**	472.9	+0.2	(-0.1 to +0.5)	0.12
**Secondary outcome**				
**Total spending on all drugs, $**	268,305	+269.8	(+44.6 to +495.1)	0.02
**Total spending on antiplatelet drugs, $**	22,486	+60.0	(+24.9 to +95.1)	<0.001

* Physicians were restricted to those who performed at least 11 diagnostic cardiac catheterizations and stent insertion procedures on Medicare beneficiaries in 2017 (N=5,730)

Supplementary Table E. Specialty of physicians in our sample

Specialty	No.	%
**Cardiology**	4,718	82.3%
**Internal Medicine**	993	17.3%
**Others**	19	0.3%
**Total**	**5,730**	100%

Supplementary Table F. Association between industry payments to physicians* for antiplatelet drugs and the use of cardiac procedures, stents, and prescriptions, for $100 increase in industry payments, restricting to interventional cardiologists

	Mean	Adjusted Difference (95% CI)		P Value
**Primary outcome**				
**Total healthcare costs for cardiac procedures, $**	53,743	+27.0	(+7.2 to +46.7)	0.01
**Number of diagnostic cardiac catheterization per cardiologist, No.**	104.6	+0.02	(-0.01 to +0.1)	0.14
**Stent use per 1,000 diagnostic cardiac catheterizations per cardiologist, No.**	475.3	+0.2	(-0.1 to +0.6)	0.13
**Secondary outcome**				
**Total spending on all drugs, $**	285,558	+250.2	(+18.6 to +481.9)	0.03
**Total spending on antiplatelet drugs, $**	23,183	+52.9	(+19.4 to +86.4)	0.002

* Physicians were restricted to cardiologists who performed at least 11 diagnostic cardiac catheterizations and performed at least 11 stent insertion procedures on Medicare beneficiaries in 2017 (N=4,718)

Supplementary Table G. Association between industry payments to physicians* for antiplatelet drugs and the use of cardiac procedures, stents, and prescriptions, for $100 increase in industry payments, restricting to interventional cardiologists who received industry payments for antiplatelet drugs

	Mean	Adjusted Difference (95% CI)		P Value
**Primary outcome**				
**Total healthcare costs for cardiac procedures, $**	55,272	+27.6	(+8.5 to +46.7)	0.005
**Number of diagnostic cardiac catheterization per cardiologist, No.**	107.3	+0.02	(-0.01 to +0.1)	0.11
**Stent use per 1,000 diagnostic cardiac catheterizations per cardiologist, No.**	469.0	+0.3	(-0.1 to +0.6)	0.11
**Secondary outcome**				
**Total spending on all drugs, $**	332,554	+170.5	(-48.6 to +389.6)	0.13
**Total spending on antiplatelet drugs, $**	29,135	+40.7	(+11.4 to +70.1)	0.01

* Physicians were restricted to cardiologists who received industry payments for antiplatelet drugs in 2016 and performed at least 11 diagnostic cardiac catheterizations and stent insertion procedures on Medicare beneficiaries in 2017 (N=2,765)

Supplementary Table H. Association between industry payments to physicians[*] for antiplatelet drugs and the use of cardiac procedures, stents, and prescriptions, for $100 increase in industry payments, with additional adjustment for the number of stent procedures in 2015

	Mean	Adjusted Difference (95% CI)		P Value
**Primary outcome**				
**Total healthcare costs for cardiac procedures, $**	57,193	+4.4	(-5.2 to +14.1)	0.37
**Number of diagnostic cardiac catheterization per cardiologist, No.**	111.1	-0.01	(-0.04 to +0.01)	0.27
**Stent use per 1,000 diagnostic cardiac catheterizations per cardiologist, No.**	472.6	+0.2	(-0.1 to +0.5)	0.13
**Secondary outcome**				
**Total spending on all drugs, $**	304,970	+226.0	(-0.4 to +452.4)	0.050
**Total spending on antiplatelet drugs, $**	24,812	+47.3	(+14.1 to +80.5)	0.01

[*] Physicians were restricted to cardiologists who performed at least 11 diagnostic cardiac catheterizations in 2017 and performed at least 11 stent insertion procedures on Medicare beneficiaries in 2017 and 2015 (N= 4,046)

Supplementary Table I. Association between industry payments to physicians* for antiplatelet drugs and the use of cardiac procedures, stents, and prescriptions, for $100 increase in industry payments, with additional adjustment for the number of antiplatelet drug prescription in 2015

	Mean	Adjusted Difference (95% CI)		P Value
**Primary outcome**				
**Total healthcare costs for cardiac procedures, $**	53,743	+15.0	(-5.0 to +35.0)	0.14
**Number of diagnostic cardiac catheterization per cardiologist, No.**	104.6	+0.003	(-0.03 to +0.04)	0.84
**Stent use per 1,000 diagnostic cardiac catheterizations per cardiologist, No.**	475.3	+0.2	(-0.1 to +0.6)	0.15
**Secondary outcome**				
**Total spending on all drugs, $**	285,558	+72.4	(-100.9 to +245.7)	0.41
**Total spending on antiplatelet drugs, $**	23,183	+14.3	(-0.3 to +28.9)	0.055

* Physicians were restricted to cardiologists who performed at least 11 diagnostic cardiac catheterizations and stent insertion procedures on Medicare beneficiaries in 2017 (N=4,718).

Supplementary Table J. Association between industry payments to physicians* for antiplatelet drugs and the use of cardiac procedures, stents, and prescriptions, for $100 increase in industry payments, with additional adjustment for antiplatelet drug prescribing costs in 2015

	Mean	Adjusted Difference (95% CI)		P Value
**Primary outcome**				
**Total healthcare costs for cardiac procedures, $**	53,743	+15.5	(-4.5 to +35.5)	0.13
**Number of diagnostic cardiac catheterization per cardiologist, No.**	104.6	+0.005	(-0.03 to +0.04)	0.78
**Stent use per 1,000 diagnostic cardiac catheterizations per cardiologist, No.**	475.3	+0.2	(-0.1 to +0.6)	0.15
**Secondary outcome**				
**Total spending on all drugs, $**	285,558	+99.9	(-76.9 to +276.8)	0.27
**Total spending on antiplatelet drugs, $**	23,183	+19.8	(+3.4 to +36.2)	0.02

* Physicians were restricted to cardiologists who performed at least 11 diagnostic cardiac catheterizations and stent insertion procedures on Medicare beneficiaries in 2017 (N=4,718).

Supplementary Table K. Association between industry payments to physicians* for antiplatelet drugs and the use of cardiac procedures, stents, and prescriptions, for $100 increase in industry payments, using negative binomial regression models

	Mean	Adjusted Difference (95% CI)		P Value
**Primary outcome**				
**Total healthcare costs for cardiac procedures, $**	53,743	+0.1%	(+0.01% to +0.1%)	0.01
**Number of diagnostic cardiac catheterization per cardiologist, No.**	104.6	+0.02%	(-0.02% to +0.1%)	0.33
**Stent use per 1,000 diagnostic cardiac catheterizations per cardiologist, No.**	475.3	+0.04%	(+0.02% to +0.1%)	0.003
**Secondary outcome**				
**Total spending on all drugs, $**	285,558	+0.1%	(+0.1% to +0.2%)	<0.001
**Total spending on antiplatelet drugs, $**	23,183	+0.2%	(+0.2% to +0.2%)	<0.001

* Physicians were restricted to cardiologists who performed at least 11 diagnostic cardiac catheterizations and stent insertion procedures on Medicare beneficiaries in 2017 (N=4,718).

Supplementary Table L. Association between industry payments to physicians* for antiplatelet drugs and the use of cardiac procedures, stents, and prescriptions, for $100 increase in industry payments, without adjustment for average HCC risk score

	Mean	Adjusted Difference (95% CI)		P Value
**Primary outcome**				
**Total healthcare costs for cardiac procedures, $**	53,743	+24.6	(+5.3 to +43.9)	0.01
**Number of diagnostic cardiac catheterization per cardiologist, No.**	104.6	+0.02	(-0.01 to +0.1)	0.24
**Stent use per 1,000 diagnostic cardiac catheterizations per cardiologist, No.**	475.3	+0.3	(-0.1 to +0.6)	0.12
**Secondary outcome**				
**Total spending on all drugs, $**	285,558	+217.3	(-24.1 to +458.6)	0.08
**Total spending on antiplatelet drugs, $**	23,183	+51.0	(+17.0 to +85.0)	0.003

* Physicians were restricted to cardiologists who performed at least 11 diagnostic cardiac catheterizations and stent insertion procedures on Medicare beneficiaries in 2017 (N=4,718).

Supplementary Table M. Association between industry payments to physicians* for antiplatelet drugs and the use of cardiac procedures, stents, and prescriptions, for $100 increase in industry payments, using physicians' practice years as a continuous variable

	Mean	Adjusted Difference (95% CI)		P Value
**Primary outcome**				
**Total healthcare costs for cardiac procedures, $**	53,743	+26.8	(+7.0 to +46.6)	0.01
**Number of diagnostic cardiac catheterization per cardiologist, No.**	104.6	+0.02	(-0.01 to +0.1)	0.14
**Stent use per 1,000 diagnostic cardiac catheterizations per cardiologist, No.**	475.3	+0.2	(-0.1 to +0.6)	0.13
**Secondary outcome**				
**Total spending on all drugs, $**	285,558	+243.9	(+8.3 to +479.6)	0.04
**Total spending on antiplatelet drugs, $**	23,183	+53.3	(+19.4 to +87.2)	0.002

* Physicians were restricted to cardiologists who performed at least 11 diagnostic cardiac catheterizations and stent insertion procedures on Medicare beneficiaries in 2017 (N=4,718).

Supplementary Table N. Association between industry payments to physicians* for antiplatelet drugs and the use of cardiac procedures, stents, and prescriptions, by industry payment category

Payment category	Adjusted difference, $ (95% CI)		P Value
**Total healthcare costs for cardiac procedures, $**			
**$0**		**Reference**	
**$1-$50**	-767	(-3,685 to +2,150)	0.61
**$51-$100**	-1,351	(-4,760 to +2,058)	0.44
**$101-$200**	+2,194	(-1,310 to +5,697)	0.22
**>$200**	+6,878	(+2,987 to +10,769)	<0.001
**Number of diagnostic cardiac catheterization per cardiologist, No.**			
**$0**		**Reference**	
**$1-$50**	-2.5	(-7.8 to +2.8)	0.35
**$51-$100**	-2.2	(-8.6 to +4.1)	0.49
**$101-$200**	+2.7	(-3.5 to +8.9)	0.39
**>$200**	+10.4	(+3.5 to +17.2)	0.003
**Stent use per 1,000 diagnostic cardiac catheterizations per cardiologist, No.**			
**$0**		**Reference**	
**$1-$50**	+8.4	(-12.7 to +29.5)	0.43
**$51-$100**	-2.5	(-24.5 to +19.5)	0.83
**$101-$200**	+7.2	(-17.0 to +31.4)	0.56
**>$200**	+10.2	(-16.1 to +36.5)	0.45
**Total spending on all drugs, $**			
**$0**		**Reference**	
**$1-$50**	+35,827	(+13,106 to +58,548)	0.002
**$51-$100**	+63,733	(+37,182 to +90,284)	<0.001
**$101-$200**	+92,850	(+67,368 to +118,332)	<0.001
**>$200**	+140,815	(+111,916 to +169,714)	<0.001
**Total spending on antiplatelet drugs, $**			
**$0**		**Reference**	
**$1-$50**	+3,842	(+1,830 to +5,854)	<0.001
**$51-$100**	+9,663	(+7,044 to +12,282)	<0.001
**$101-$200**	+11,382	(+8,666 to +14,098)	<0.001
**>$200**	+24,799	(+20,550 to +29,048)	<0.001

* Physicians were restricted to cardiologists who performed at least 11 diagnostic cardiac catheterizations and stent insertion procedures on Medicare beneficiaries in 2017 (N=4,718).

Supplementary Table O. Association between industry payments to physicians* for antiplatelet drugs in 2017 and the use of cardiac procedures, stents, and prescriptions in 2017 (for $100 increase in industry payments)

	Mean	Adjusted Difference (95% CI)		P Value
**Primary outcome**				
Total healthcare costs for cardiac procedures, $	37,912	+126.3	(+1.9 to +250.7)	0.047
Number of diagnostic cardiac catheterization per cardiologist, No.	79.0	+0.2	(-0.02 to +0.4)	0.08
Stent use per 1,000 diagnostic cardiac catheterizations per cardiologist, No.	300.7	+1.3	(-0.1 to +2.7)	0.07
**Secondary outcome**				
Total spending on all drugs, $	302,341	+264.8	(-369.3 to +898.9)	0.41
Total spending on antiplatelet drugs, $	19,474	+95.9	(-20.9 to +212.7)	0.11

* Physicians were restricted to cardiologists who performed at least 11 diagnostic cardiac catheterizations on Medicare beneficiaries in 2017 (N=7,456)
